# The Trade-Off between Accuracy and Accessibility of Syphilis Screening Assays

**DOI:** 10.1371/journal.pone.0075327

**Published:** 2013-09-16

**Authors:** Pieter W. Smit, David Mabey, John Changalucha, Julius Mngara, Benjamin Clark, Aura Andreasen, Jim Todd, Mark Urassa, Basia Zaba, Rosanna W. Peeling

**Affiliations:** 1 Department of Infectious & Tropical diseases, London School of Hygiene & Tropical Medicine, London, United Kingdom; 2 National Institute for Medical Research, Mwanza, Tanzania; 3 Mwanza intervention Trials Unit, Mwanza, Tanzania; University of Kentucky College of Medicine, United States of America

## Abstract

The availability of rapid and sensitive methods to diagnose syphilis facilitates screening of pregnant women, which is one of the most cost-effective health interventions available. We have evaluated two screening methods in Tanzania: an enzyme immunoassay (EIA), and a point-of-care test (POCT). We evaluated the performance of each test against the *Treponema pallidum* particle agglutination assay (TPPA) as the reference method, and the accessibility of testing in a rural district of Tanzania. The POCT was performed in the clinic on whole blood, while the other assays were performed on plasma in the laboratory. Samples were also tested by the rapid plasma Reagin (RPR) test. With TPPA as reference assay, the sensitivity and specificity of EIA were 95.3% and 97.8%, and of the POCT were 59.6% and 99.4% respectively. The sensitivity of the POCT and EIA for active syphilis cases (TPPA positive and RPR titer ≥1/8) were 82% and 100% respectively. Only 15% of antenatal clinic attenders in this district visited a health facility with a laboratory capable of performing the EIA. Although it is less sensitive than EIA, its greater accessibility, and the fact that treatment can be given on the same day, means that the use of POCT would result in a higher proportion of women with syphilis receiving treatment than with the EIA in this district of Tanzania.

## Introduction

The prevalence of syphilis is high among pregnant women attending antenatal clinics in sub-Saharan Africa [1]. Syphilis in pregnancy can have devastating effects on the developing fetus and is a major cause of stillbirths and neonatal deaths in Africa [2]. Screening with an adequate diagnostic test and treatment of pregnant women with a single dose of benzathine penicillin before the third trimester could prevent more than 300, 000 stillbirths and neonatal deaths annually [3,4].

Latent syphilis can only be diagnosed serologically. Laboratory based assays such as the *Treponema pallidum* particle agglutination assay (TPPA) and rapid plasma reagin (RPR) test are widely used. As the agglutination is interpreted by a technician, the test result is subjective. Enzyme immunoassays (EIA) are now recommended for syphilis screening in Europe [5]. They are easy to use, provide objective results, and are well adapted to high throughput laboratories; but they are more expensive than the other assays, require equipment (a plate washer and a plate reader), and as TPPA and RPR, require cold storage of consumables, which is a limiting factor for some settings [6,7].

Most of the available syphilis point-of-care tests (POCT) are lateral flow based treponemal tests that provide a result in 10-30 minutes and do not require any equipment. In contrast to these laboratory based assays mentioned above, POCTs are easy to perform, require only a drop of blood collected by finger prick, and do not require refrigeration; they could enable same day testing and treatment for syphilis at any health facility, but are less sensitive than laboratory based assays [8].

Selecting a screening assay, particularly in an African country, should not be solely based on the performance of the assay. Besides the performance of a test, necessary equipment, cold chain requirements, and complexity of executing tests should be taken into consideration. Selecting a screening assay is therefore often a trade-off between the performance of the assays, cost, and accessibility for patients to be screened. Comparative studies have been performed [9-12] but, to our knowledge, evaluations that include performance and accessibility of syphilis screening tests have not been published previously. This study evaluates two screening assays, a POCT and an EIA, to review the trade-off between performance and accessibility of syphilis assays used to screen pregnant women in an African district.

## Methods

### Samples and Field Procedures

The study was approved by the ethical review committee of the National Institute for Medical Research (NIMR) in Tanzania and the ethical committee of the London School of Hygiene and Tropical Medicine. The Kisesa open cohort is a well-established on-going community-based study in Northern Tanzania [13,14]. The cohort study uses regular demographic surveillance with serological surveys, providing data on HIV incidence and prevalence [15]. Regular demographic surveillance ensures representation of all members of the population, with serological surveys providing data on HIV prevalence and incidence [15]. Subjects that accepted voluntary counseling and testing (VCT) were tested for HIV and syphilis using POCT performed by trained clinicians. All subjects with a positive syphilis result were given free medical treatment according to Tanzanian government guidelines, and all those positive for HIV were referred to the Tanzanian care and treatment center.

Whole blood was collected by venipuncture into heparinized tubes from consenting subjects, and transported to the NIMR laboratory in Mwanza. Within 24 hours the blood was centrifuged and stored at -20°C. Samples were collected from April 2010 until September 2010 and were tested until March 2011. Samples were bar-coded to ensure anonymous testing. Double data entry was used to enter the results. Results were entered automatically (EIA) or manually into the Laboratory Information Management System (LIMS).

### Access to screening

We visited all 50 health facilities in one district of Mwanza Region (Geita District), to ask how many women attended each antenatal clinic per month. Geita District has one district hospital, 8 health centers, which tend to be in larger villages, and 41 rural dispensaries.

### Point-of-care test

The SD bioline syphilis 3.0 POCT (Standard Diagnostics, Kyong gi-do, Korea) was included in this study as it was found to have acceptable performance (according to the WHO ASSURED criteria) [16]. Additionally, the SD bioline POCT was made available through the WHO Bulk Procurement Programme and has been previously used in Tanzania [17]. POCT testing was done by trained clinicians who were contracted and trained by the Kisesa open cohort study. The POCT was performed with whole blood samples collected by finger prickA timer was used to ensure that the test was read after exactly 15 minutes. The manufacturer’s instructions were followed.

### TPPA

A total of 2099 plasma aliquots were raised to room temperature and tested by TPPA (Fujirebio, Tokyo, Japan). TPPA was performed according to the manufacturer’s instructions. TPPA results were read by two trained and experienced technicians. The reading of the TPPA occurred while masked to results of other tests. Discordant results between the two technicians were discussed and one outcome was agreed by consensus. Results were deemed indeterminate for biologically reactive samples or when a conclusive outcome could not be obtained due to difficulty in interpretation or lack of technician’s agreement.

### Enzyme ImmunoAssay

The Syphilis Enzyme ImmunoAssay (EIA) (Lab21 healthcare, Kentford, UK) became available at the midpoint of the study and was performed on 1041 samples (49.6%). It was performed according to the manufacturer’s instructions and read by Optical Density (OD) 450/620nm using an automated reader (DTX 800, Beckman Coulter, USA) which calculated the cut-off according to the instruction manual.

### RPR

Quantitative Rapid plasma Reagin (RPR) (BD Macro-vue RPR, Beckton Dickinson, Sparks MD, USA) was performed on all samples according to the manufacturer’s instructions. An active syphilis infection was defined as TPPA positive and RPR titer ≥1/8 [18].

Sensitivities and specificities, and exact binomial confidence intervals are given (diagt function in Stata). The agreement between various methods was tabulated. Microsoft Excel (Microsoft Corp., USA) and the statistical package Stata 11 (Stata corp LP, Texas, USA) were used to analyze the results.

## Results

### Point- of- care test

The POCT was evaluated with a set of 2099 samples. Of these, 17.1% of samples were positive by TPPA and 10.7% by POCT. With TPPA as reference, the POCT had 11 false positive and 145 false negative results, giving a sensitivity of 59.6% (95% confidence interval CI: 54.3-64.7%) and specificity of 99.4% (95% CI: 98.9-99.7%) (Table 1). There was a 92.6% agreement between POCT and TPPA.

**Table 1:** The TPPA and POCT performances are given with RPR results divided into titres lower (<1/8) or higher than 1/8 (≥1/8).

**TPPA**	**POCT**	**RPR**			**Samples**
		<1/8	≥1/8	Neg	
**+**	+	71	41	102	214
**+**	-	21	9	115	145
**-**	+	3	0	8	11
**-**	-	44	9	1676	1729
Total		**139**	**59**	**1901**	**2099**

+ positive, - negative, Neg=negative

Of the 145 false negatives by POCT, 115 (79%) were RPR negative and 31 were RPR positive. Fifty out of 2099 samples tested by POCT had active syphilis (positive TPPA and RPR titre ≥ 1/8), of which 41 were detected by POCT, giving a sensitivity of 82% (69.2%-90.2%) and specificity of 100%.

### EIA

The EIA was evaluated with a subset of 1041 samples that were available at the second half of the study. Of these, 18.1% of samples were positive by TPPA, 11.5% by POCT and 19.1% by EIA.

With TPPA as reference, the EIA had 20 false positive and 9 false negative results, giving a sensitivity of 95.2% (95% CI: 91.1-97.8%) and specificity of 97.7% (95% CI: 96.4-98.6%) (Table 2). There was a 97.3% agreement between TPPA and EIA. The EIA showed a sensitivity and specificity of 100% to detect active syphilis cases (Table 2).

**Table 2:** The TPPA and EIA performances are given with RPR results divided into titres lower (<1/8) or higher than 1/8 (≥1/8).

**TPPA**	**EIA**	**RPR**			**Samples**
		<1/8	≥1/8	Neg	
**+**	+	53	26	100	179
**+**	-	0	0	9	9
**-**	+	3	0	17	20
**-**	-	30	4	799	833
Total		**86**	**30**	**925**	**1041**

+ positive, - negative, Neg=negative

All 29 samples for which a discordant result was obtained by the two laboratory methods were retested. Although retesting discordant results provides little certainty regarding the true syphilis status of the patients, it is informative to review the reproducibility of the tests. On retesting, 8 out of 9 TPPA positive samples became negative and one became TPPA indeterminate, and 6 out of 20 TPPA negative samples became positive. When retesting the samples with discordant results, only four EIA positive samples changed outcome (4 initially positive samples became negative out of 29 discordant results)

### Comparison of EIA and POCT

**Figure 1:**
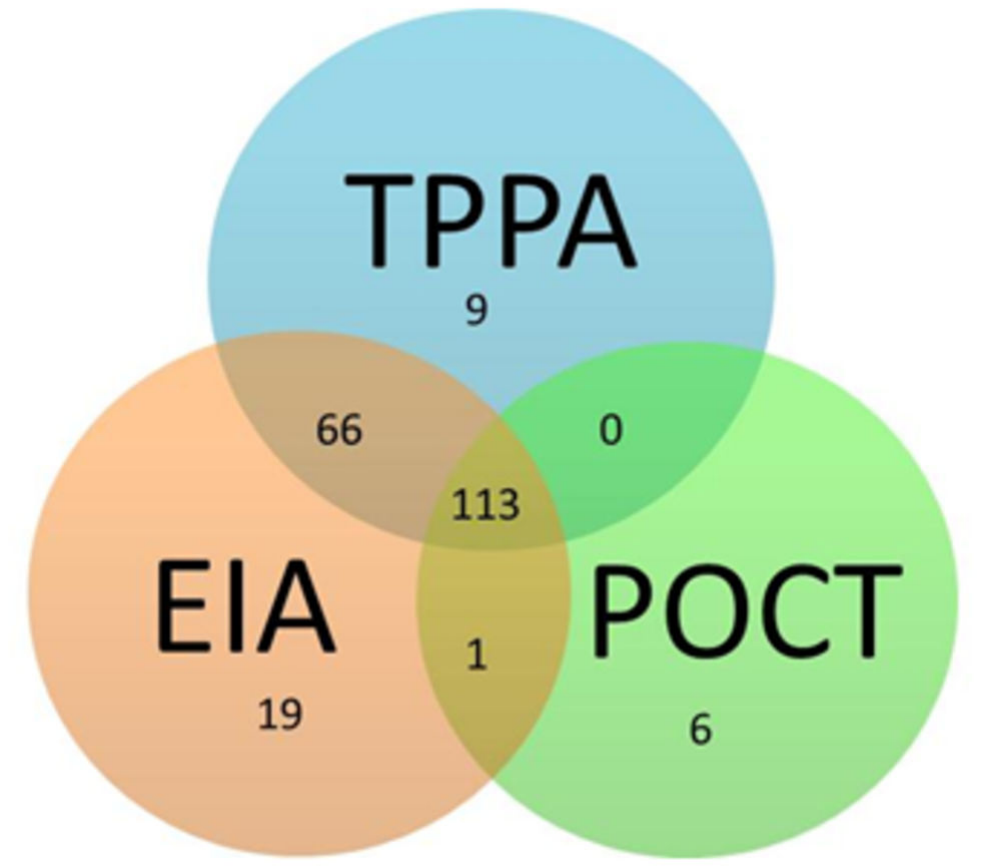
Distribution of positive samples among the three assays. Numbers represents positive samples detected by three assays (number is given in 3 circles), or by two assays (overlapped by two circles) or by one assay (given as number below the assay name). 214 positives detected by any of the three methods out of 1041 samples.

The performance of the EIA and POCT were compared with the samples that were tested by EIA, TPPA and POCT. Figure 1 shows the distribution of 214 positive out of 1041 samples identified by one or more of the screening assays.

### Accessibility

Syphilis screening is essential for antenatal care and we therefore decided to assess the accessibility of these syphilis tests in antenatal care clinics. Geita District has one district hospital, 8 health centers, which tend to be in larger villages, and 41 rural dispensaries. The EIA could only be performed at the district hospital, since health centers and dispensaries do not have centrifuges for plasma separation, cold storage capabilities, plate washers or readers. The POCT could successfully be implemented in the district hospital, health centers and dispensaries. On average, 517 pregnant women attend the district hospital ANC for the first time per month, whereas 1164 attend a health center and 1685 a dispensary. If the prevalence of active syphilis is 2.3%, as found in this study, 77 pregnant women with active syphilis would be expected to attend an ANC in Geita District each month. Twelve of these would be attending the district hospital, and 65 would be attending health centers or dispensaries. If POCT wereused at the ANC clinics (including the district hospital), with a sensitivity of 82%, it would detect 63 (82%) pregnant women and enable them to be offered immediate treatment, whereas without the POCT only 12 of 77 pregnant women with syphilis (16%) would be identified.

## Discussion

To our knowledge, this is the first study comparing POCT and EIA performed in an African setting. Additionally, this is the first study that includes both the accessibility and diagnostic performances of syphilis screening assays into one evaluation.

It Is Important to Note That This Study Was Performed in Tanzania on Tanzanian Samples, Which Potentially Influences the Results. African Samples Can Contain High Immunoglobulin Levels Due to Other Infections Which Potentially Cause False Positive Test Results. This Is Particularly the Case for RPR Tests, Which Can Cause Biologically False Positive Results in HIV Positive Patients [19]. 

[20-22]. Although tests were performed and stored according to the manufacturer’s recommendations, potential environmental effects by transporting and using the kits under tropical conditions could not be completely ruled out. Performing tests in an African country relies more heavily on the robustness of the tests than in developed countries. This could potentially influence the test results. The need for quality control and appropriate training is important for any diagnostic test whether it is laboratory based or POCT. Most quality assurance methods are not suitable for monitoring POCT that are performed by healthcare workers at remote locations, making it more challenging to assure the quality of POC testing [17,23].

The reproducibility of the EIA evaluated in this study was good, with few samples giving discordant results on re-testing. The samples that were discordant had very low ODs, just above the cut-off. Therefore, EIA seems to be a more stable and reliable test compared to TPPA.

Adverse pregnancy outcomes due to syphilis are seen in women with RPR titers of ≥ 1:8 [2]. In this study, the EIA had a higher sensitivity for the detection of these cases than the POCT (100% versus 82%), but a major limiting factor is that the EIA can only be performed when sufficient laboratory infrastructure is available. Most importantly, in Mwanza region, the POCT would enable more pregnant women with syphilis to be identified due to its greater accessibility, since pregnant women can be screened not only at the hospital but also at health centers and dispensaries. The RPR test is easier to perform than the EIA and requires less equipment, but requires access to a laboratory with electricity. It has been used to screen pregnant women in many developing countries, but results obtained in rural health facilities have not been encouraging [24,25]. Therefore, we did not evaluate the option of on- or off-site RPR & TPPA screening in Mwanza region In some countries blood is taken from pregnant women at rural health facilities and sent to a central laboratory to be tested for syphilis (RPR and TPPA/TPHA), but under these circumstances women with syphilis will only be treated if they return for their results [26]. A study in Kenya found that less than 10% of pregnant women with syphilis received appropriate treatment when serological tests were performed at a central laboratory [27]. When same day testing and treatment were made available in these clinics, more than 90% of infected women were treated [28].

A test which gives a result in 15 minutes, allowing patients to be tested and treated on the same day, may result in more cases being treated than a more sensitive laboratory test which requires patients to return for treatment at a later date. The great advantage of the new POCTs for syphilis is that they do not require laboratory equipment or electricity, and can make same day testing and treatment for syphilis available at any health facility [17,29]. Based on the performance and accessibility of both tests, the POCT is the best option for this district in Tanzania and potentially for other settings in developing countries as well.
